# Penetrating eyelid injury: a case report and review of literature

**DOI:** 10.1186/1746-160X-5-2

**Published:** 2009-01-14

**Authors:** Ehab Wasfi, B Kendrick, T Yasen, Priya Varma, Alaa A Abd-Elsayed

**Affiliations:** 1 Department of Ophthalmology, Accident and Emergency Department, Great Western Hospital, Swindon, UK; 2 Anesthesiology Outcomes Research, Anesthesiology Department, Cleveland Clinic, Cleveland, USA; 3 Public Health and Community Medicine Department, Faculty of Medicine, Assiut University, Assiut, Egypt

## Abstract

**Introduction:**

In literature, many different types of foreign objects have been found to have caused eye injuries. These objects can range from organic to inorganic matter such as glass, wood, pencil, nails and fishhooks. Once the injury is recognized, removal of the foreign body and technique used in the management of the injury is very important to reduce further ocular damage. This case report investigates an injury caused by an object similar to a fishhook that pierced into the eyelid in the opposite direction to normal.

**Case presentation:**

A 19 year old man presented with a one hour history of the right upper eyelid injury from a wire fence. The loose end of the wire penetrated the full thickness of the eyelid in the direction opposite to the normal. The wire passed from under the eyelid, through the centre of the upper lid, to the external surface. After the application of topical anesthetic drops, the eye could be opened manually, the lid averted, and the wire passed out through the defect. No complications were observed. Post removal, the acuity increased to 6/9 and there was no intraocular penetration. Full recovery was observed as well.

**Conclusion:**

A severe eyelid penetrating injury can be uncomplicated with a full recovery when there is no intraocular penetration. It is also possible to have an injury pass under the lower margin of the lid and penetrate from inside to out, with no associated corneal injury.

## Introduction

Orbital injury may be caused by several types of foreign bodies such as organic and inorganic matter, non-autogenous surgical implants and allograft, and surgical hardware and materials utilized in reconstructive surgery. In eye injury patients, the nature of the foreign body determines the clinical behavior; inert objects such as steel and glass may not cause significant inflammation to warrant their removal. Removal of organic foreign bodies, however, is mandatory since these objects usually lead to secondary infection [1].

Once the injury has occurred, the eye should be examined very gently without putting any pressure on the globe. Prolapsed of the intraocular contents and irreversible damage can be caused if the eye and orbit are not examined carefully. Signs to look for include a distorted pupil, cataract, prolapsed black uveal tissue on the ocular surface, and vitreous hemorrhage. The pupil should be dilated (if there is no head injury) and a thorough search made for an intraocular foreign body [2].

In this case report, upper eyelid injury is of interest. Ocular fishhook injuries can cause potentially devastating ocular trauma. *Aiello et al *reported five cases of penetrating ocular fishhook injuries and showed that with appropriated surgical techniques excellent visual outcome can be achieved in these cases. Appropriate techniques have to be employed to remove the fishhook and avoid major damage to the eyelid anatomy [3].

## Case Presentation

**Figure 1:**
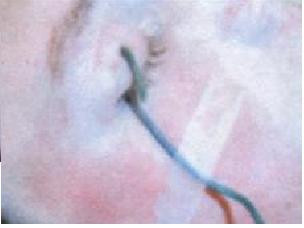
**Patient eye on arrival**.

A 19 year old man presented to the Accident and Emergency Department with a one hour history of the right upper eyelid injury from a wire fence, (figure 1). The patient was walking across an allotment when he fell onto a damaged fence. The loose end of the wire penetrated the full thickness of the right upper eyelid. The patient was unable to extricate himself, requiring the Fire Brigade to cut him free. Of relevant past history, there was an injury to the same eyelid from a coat hanger two years earlier.

Upon gentle examination with no external pressure, the patient was unable to open the eye himself. The wire passed from under the eyelid, through the centre of the upper lid, to the external surface. Approximately 15 mm of wire was superficial to the lid margin; the cut end was approximately 90 mm and taped to the cheek for security.

The patient had eaten ninety minutes previously so he was unfit for a general anesthetic. The decision was made to infiltrate with local anesthetic and remove the foreign body. This was complicated by the patient's inebriation and needle phobia.

**Figure 2:**
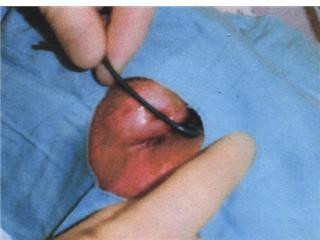
**Removal of the wire from the patients' eyelid**.

**Figure 3:**
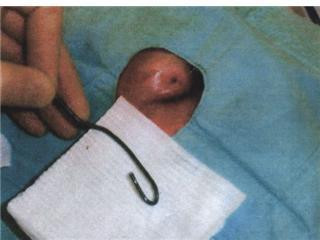
**The wire completely removed from the eyelid**.

**Figure 4:**
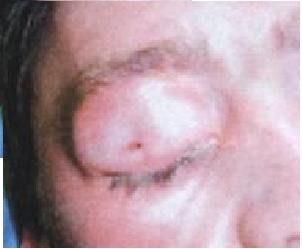
**Patients eye after complete removal of the wire**.

1% Lignocaine was infiltrated in to the upper lid, the lid averted, and the wire passed out through the defect, (figure 2 and 3). Post removal, the acuity increased to 6/9 and there was no intraocular penetration, (figure 4). No abnormalities were detected in the anterior or posterior segments and intraocular pressure was within the normal range.

After the application of topical anesthetic drops, the eye could be opened manually. Acuity in the right was counting fingers. The anterior chamber was formed and there were no pupil abnormalities. It was difficult to assess whether there was any intraocular penetration. In addition, the injured region was examined for any remains or other possible foreign bodies.

At follow up the next day acuity was still maintained and one week later, after a full course of antibiotics, examination was unremarkable with equal acuity bilateral

## Discussion

A variety of orbital foreign bodies have been reported in the literature to have penetrated the eyelids. These include glass, stone, metal, wood, graphite, button, faucet handle, fish jaw, iron hat peg, chopstick, pencil, large wooden plank, pocket knife, meat hook, and pitchfork [4]. Furthermore, removal of such foreign bodies and the appropriate technique used is important in the management of the injury otherwise it could lead to vision loss, corneal scaring, retinal detachment and endophthalmitis [5].

A review of the appropriate literature demonstrated that penetrating eyelid injury, particularly from fishhooks, was common, with a range of removal techniques available such as retrograde, needle cover, advance and cut, string yank and vertical eyelid-splitting [5]. There were no reports found of penetration from anything with a greater caliber or injuries which penetrated in the opposite direction to normal.

The unusual aspects of this presentation were firstly the nature of the injury, in that it was sustained from a fall on to a sharp object rather than a moving foreign body. Secondly there was enough force to penetrate the lid but essentially left the globe without injury. Lastly, the direction of the penetration was unusual as it passed from the under to the external surface.

Moreover, topical anesthesia has been shown to be safe and effective [6] especially in this case where the patient had expressed needle phobia. As a result, the decision to infiltrate local anesthetic is more appropriate as the advantages of local anesthesia include immediate onset, short duration of action, rapid return of visual function, and avoidance of the attendant risks of general anesthesia. These advantages determine a shorter hospital stay, more rapid resumption of a regular diet and normal insulin or oral therapy, and ambulation for the patient [7]. Plus, it has been found that this method of anesthesia is particularly useful in patients who have distressing fears of injection and in whom poor cooperation renders the patient vulnerable to needle related injuries [8].

In addition to removing the fishhook, post- removal wound care is also of interest. After removal of the fishhook, the wound should be explored for possible foreign bodies. It is usually sufficient to leave the wound open, and then apply an antibiotic ointment and a simple dressing. Tetanus toxoid should be administered to persons for whom more than five years has elapsed since their last tetanus booster [9]. In this case, the patient had a recent history (less than five years) of tetanus booster as a result; he did not require a tetanus shot.

This case demonstrates that a severe eyelid penetrating injury can be uncomplicated with a full recovery when there is not intraocular penetration. It is also possible to have an injury pass under the lower margin of the lid and penetrate from inside to out, with no associated corneal injury.

## Conclusion

A severe eyelid penetrating injury can be uncomplicated with a full recovery when there is no intraocular penetration. It is also possible to have an injury pass under the lower margin of the lid and penetrate from inside to out, with no associated corneal injury.

## Consent

Written informed consent was obtained from our patient for publication of this case report and the accompanying images. A copy of the written consent is available for review by the Editor-in-Chief of this journal.

## Competing interests

The authors declare that they have no competing interests.

## Authors' contributions

EW, BK, TY carried out the patient diagnosis, investigation, follow up and management. PV participated in writing the final manuscript. AAA-E participated in patient management, general coordination, drafting of the manuscript, writing the final manuscript and provided important suggestions.

All authors read and approved the final manuscript.

## References

[B1] Karcioglu Z, Nasr A (1998). Diagnosis and management of orbital inflammation and infections secondary to foreign bodies: a clinical review. Orbit Opthalmology.

[B2] Khaw P, Shah P, Elkington A (2004). ABC of eyes, injury to the eye. BMJ.

[B3] Srinivasan S, Macleod S (2001). Fish hook injury to the eyelid. Indian J Ophthalmol.

[B4] Liu D, Al Shail E (2002). Retained orbital wooden foreign body a surgical technique and rationale. Ophthalmology.

[B5] Fuentes-Mallozzi D, Méndez-Orozco C (2005). Eyelid fish-hook injury: case report. Bol Med Hosp Infant Mex.

[B6] Karp C, Cox T, Wagoner D, Ariyasu R, Jacobs S (2001). Intracameral anesthesia: a report by the American Academy of Ophthalmology. American Academy of Ophthalmology.

[B7] Boscia F, La Tegola M, Columbo G, i Alessio G, Sborgia C (2003). Combined topical anesthesia and sedation for open-globe injuries in selected patients. American Academy of Ophthalmology.

[B8] Li R, Lai J, Ng J, Law R, Lau E, Lam D (2003). Efficacy of Lignocaine 2% gel in chalazion surgery. British J of Opthalmol.

[B9] Gammons M, Jackson E (2001). Fish hook removal. Am Fam Physician.

